# Socio-ecological factors shape the distribution of a cultural keystone species in Malaysian Borneo

**DOI:** 10.1038/s44185-022-00008-w

**Published:** 2023-02-17

**Authors:** David J. Kurz, Thomas Connor, Jedediah F. Brodie, Esther L. Baking, Sabrina H. Szeto, Andrew J. Hearn, Penny C. Gardner, Oliver R. Wearn, Mairin C. M. Deith, Nicolas J. Deere, Ahmad Ampeng, Henry Bernard, Jocelyn Goon, Alys Granados, Olga Helmy, Hong-Ye Lim, Matthew Scott Luskin, David W. Macdonald, Joanna Ross, Boyd K. Simpson, Matthew J. Struebig, Jayasilan Mohd-Azlan, Matthew D. Potts, Benoit Goossens, Justin S. Brashares

**Affiliations:** 1 Department of Environmental Science, Policy, and Management, University of California, Berkeley, Berkeley, CA 94720 USA; 2 Environmental Science Program, Trinity College, Hartford, CT 06106 USA; 3 Division of Biological Sciences & Wildlife Biology Program, University of Montana, Missoula, MT 59812 USA; 4 Institute of Biodiversity and Environmental Conservation, Universiti Malaysia Sarawak, 94300 Kota Samarahan, Sarawak Malaysia; 5 Institute for Tropical Biology and Conservation, Universiti Malaysia Sabah, 88450 Kota Kinabalu, Sabah Malaysia; 6 School of the Environment, Yale University, New Haven, CT 06511 USA; 7 Sabrina Szeto Consulting, Isen, Bavaria 84424 Germany; 8 Wildlife Conservation Research Unit, Department of Biology, The Recanati-Kaplan Centre, University of Oxford, Oxford, UK; 9 RSPB UK Headquarters, The Lodge, Sandy, Bedfordshire SG19 2DL UK; 10 Danau Girang Field Centre, c/o Sabah Wildlife Department, Wisma Muis, Kota Kinabalu, Sabah Malaysia; 11 Fauna & Flora International, Vietnam Programme, Hanoi, Vietnam; 12 Institute for the Oceans and Fisheries, Faculty of Science, Vancouver Campus, The University of British Columbia, AERL, 2202 Main Mall, Vancouver, BC V6T 1Z4 Canada; 13 Durrell Institute of Conservation and Ecology, School of Anthropology and Conservation, University of Kent, Canterbury, UK; 14 Forest Department Sarawak, Level 15, East Wing, Bangunan Baitul Makmur II, Medan Raya, Petra Jaya, 93050 Kuching, Sarawak Malaysia; 15 Felidae Conservation Fund, 100 Shoreline Hwy, Suite 100B, Mill Valley, CA 94941 USA; 16 Forever Sabah, H30, Gaya Park, Lorong Muntahan 1 C, Jalan Penampang, 88300 Kota Kinabalu, Sabah Malaysia; 17 School of Biological Sciences, University of Queensland, St. Lucia, QLD Australia; 18 Copenhagen Zoo, Department of Research & Conservation, Frederiksberg, Denmark; 19 Organisms and Environment Division, School of Biosciences, Cardiff University, Cardiff, UK; 20 Sabah Wildlife Department, Wisma Muis, Kota Kinabalu, Sabah Malaysia; 21 Sustainable Places Research Institute, Cardiff University, Cardiff, UK

**Keywords:** Biogeography, Socioeconomic scenarios, Conservation biology, Ecological modelling, Tropical ecology, Geography, Social sciences

## Abstract

Biophysical and socio-cultural factors have jointly shaped the distribution of global biodiversity, yet relatively few studies have quantitatively assessed the influence of social and ecological landscapes on wildlife distributions. We sought to determine whether social and ecological covariates shape the distribution of a cultural keystone species, the bearded pig (*Sus barbatus*). Drawing on a dataset of 295 total camera trap locations and 25,755 trap days across 18 field sites and three years in Sabah and Sarawak, Malaysian Borneo, we fitted occupancy models that incorporated socio-cultural covariates and ecological covariates hypothesized to influence bearded pig occupancy. We found that all competitive occupancy models included both socio-cultural and ecological covariates. Moreover, we found quantitative evidence supporting Indigenous pig hunting rights: predicted pig occupancy was positively associated with predicted high levels of Indigenous pig-hunting groups in low-accessibility areas, and predicted pig occupancy was positively associated with predicted medium and low levels of Indigenous pig-hunting groups in high-accessibility areas. These results suggest that bearded pig populations in Malaysian Borneo should be managed with context-specific strategies, promoting Indigenous pig hunting rights. We also provide important baseline information on bearded pig occupancy levels prior to the 2020–2021 outbreak of African Swine Fever (ASF), which caused social and ecological concerns after mass dieoffs of bearded pigs in Borneo. The abstract provided in Malay is in the Supplementary file.

## Introduction

Socio-cultural and biophysical landscapes are fundamentally connected. However, our empirical understanding of the links between them is still limited. While researchers often quantitatively examine links between biophysical factors and wildlife, far less quantitative work has been carried out on the influence of socio-cultural factors on wildlife distributions. Yet socio-cultural factors—such as ethnic identity, culturally-distinctive hunting practices, armed conflict, recreation, feasts, traditions, and value systems—have been shown to have far-reaching implications for animal behavior, wildlife distributions, and conservation efforts^1–6^. As such, social and cultural practices, tolerances, affinities, and other socio-cultural factors require more attention as important predictor variables, alongside ecological variables, for determining occurrence patterns of wildlife species^7^.

A primary challenge has been integrating nuanced quantitative measures of socio-cultural factors into wildlife distribution modeling. Recently, socio-cultural covariates have begun to move beyond broader indices of human disturbance or footprint to include culturally-shaped metrics, such as hunting accessibility, social carrying capacity for development, or religious practices^8–10^. These more recently adopted metrics reflect an emerging understanding of the conceptual complexity of human-wildlife interactions, which take place within nested social and ecological systems^11^. While the number of socio-ecological studies has increased dramatically in recent years^12^, greater attention is needed to the integration of social and ecological variables in wildlife modeling^13^. However, relatively few robust case studies have leveraged the social and environmental data needed to quantify their joint influence on species distributions.

The bearded pig, *Sus barbatus*, is an ideal species for assessing the relative contribution of socio-cultural and ecological variables to wildlife distributions. Bearded pigs are sensitive to social factors, such as hunting practices, which can influence their local distribution and behavior^6,14^. The bearded pig is also the most favored terrestrial game species for many non-Muslim Indigenous communities in Sabah and Sarawak, accounting for up to 54–97% of their wild meat^14,15^. Within these communities, the bearded pig is still hunted using both historical approaches (e.g. dogs and spear, snare, nets) and contemporary methods (e.g. on foot with a gun, drive hunt)^6,16^. Moreover, the species plays a central role in a variety of Indigenous ceremonial practices and celebrations^17^, including gifting of the meat to others, and the pig is also hunted for additional reasons, such as pest control, sport, and sale^6,16^. However, among one pool of Indigenous pig hunters, only about a quarter reported hunting bearded pigs for sale, and several hunters felt that hunting for sale was unnecessary or irresponsible^6^. In Sabah and Sarawak, the largest Indigenous pig-hunting group makes up roughly 21 and 29% of the total population of each state, respectively, accounting for a substantial proportion of the population of each state^18^.

In our study area, bearded pig hunting has been a particularly salient cultural force for thousands of years^6,19^. This hunting relationship is given further texture by the role of religion in shaping cultural affinities toward bearded pigs. In Sabah and Sarawak, religion and ethnicity are fairly closely linked. According to census data, 100% of Malays in both states are registered as Muslim, and roughly 75% of Kadazandusun-Muruts (KDM, an abbreviation used locally; KDMs are the primary pig-hunting Indigenous group in Sabah) and Ibans (the primary pig-hunting Indigenous group in Sarawak) identify as Christians^18^. Wild pork is highly favored by Christian KDM and Iban communities in Sabah and Sarawak^6,14^, but pork is actively avoided in Muslim communities in Malaysia generally^20^. Therefore, the role of bearded pig hunting in contemporary Malaysian Borneo also speaks to the role of religion in shaping socio-ecological interactions. Given these dynamics as well as the potential threat to pig populations posed by hunting^14,21^, it is critical to better understand how ethnicity, and religious factors tied to ethnicity, may be related to hunting pressure and bearded pig distributions in Malaysian Borneo. It is also critical to understand the role of landscape accessibility and population density on bearded pig distributions, as both are likely to influence hunting pressure in the region^10^. Considering the ancient history of sustained pig hunting in Sabah^22^, it is possible that hunting practices in some areas may be neutral or positively associated with pig occupancies. However, with modern hunting techniques and extensive land-use change in Sabah and Sarawak, bearded pigs have experienced local declines and behavior change over time, complicating this question^6,14^.

Physical ecological factors are also likely to shape the distribution of the bearded pig. The species is dependent on forest habitat for several of its behaviors, such as wallowing, nest building, and mud scraping^23^. Additionally, its natural history is closely linked to Southeast Asian forest phenology due to its local and long-distance movements to track Dipterocarp fruit during mast fruiting events^24–27^. Loss of Dipterocarp forests has not only led to fewer reports of nomadic movements in places^6^, but has also led to an estimated 23% decline in bearded pig habitat in Borneo^28^. As forests have declined, fruit provision from Dipterocarps has been replaced in many areas by subsidies from oil palm plantations that fruit throughout most of their lifecycle^21,23,29,30^. At fine scales, bearded pigs are known to be capable of sustaining populations in heavily logged areas with oil palm fruit subsidies^23,31^, but it is unclear how forest and oil palm patchworks are shaping pig distribution at broad scales. As such, understanding the ecological correlates of pig distributions will help plan large landscape configurations that sustain healthy bearded pig populations.

Here, we integrate socio-cultural and ecological covariates to quantify their collective influence on the distribution of a cultural keystone species, the bearded pig. Specifically, we investigate the influence of two Indigenous, predominantly Christian pig-hunting groups on distributions of bearded pigs. By considering the influence of Indigenous hunting, alongside a metric of landscape accessibility and population density, we provide a link toward understanding a broader suite of socio-cultural covariates on wildlife distributions. Selecting Malaysian Borneo as our study area allowed us to investigate socio-ecological dynamics by drawing from extensive camera trap surveys, a published hunting accessibility metric, and census data for Sabah and Sarawak. Analyzing data from 295 camera locations distributed across land-use, management, and socio-cultural contexts, we investigate how social and ecological factors together shape bearded pig occupancy. We ran occupancy models in a multi-model approach with top models ranked by AICc. Our results: (a) provide empirical associations between socio-ecological factors and the distribution of a large-bodied game species; (b) provide area-specific baseline evidence of bearded pig occupancies before the 2020–2021 African Swine Fever (ASF) outbreak; and (c) quantitatively show that Indigenous hunting in our study area can be compatible with high pig occupancies. In light of these findings, we discuss the implications of our results for context-specific bearded pig management in Sabah and Sarawak.

## Results

### Overview of results

Our findings show that bearded pig distributions are associated with both social and ecological covariates in Malaysian Borneo. In particular, bearded pig occupancies are significantly negatively associated with one ecological covariate (distance to water) and with the interaction between two social covariates (pig-hunting group and hunting accessibility). In low and high accessibility areas, pig occupancy showed differing associations with percentage of the population belonging to an Indigenous pig-hunting group. In low accessibility areas, pig occupancy showed a positive association with a predicted high percentage of the population belonging to a pig-hunting group. In high accessibility areas, pig occupancy showed a positive association with a predicted medium to low percentage of the population belonging to a pig-hunting group. Additionally, estimated pig occupancies were relatively high across most study sites.

### Bearded pig occupancy associations with socio-cultural and ecological factors

**Fig. 1:**
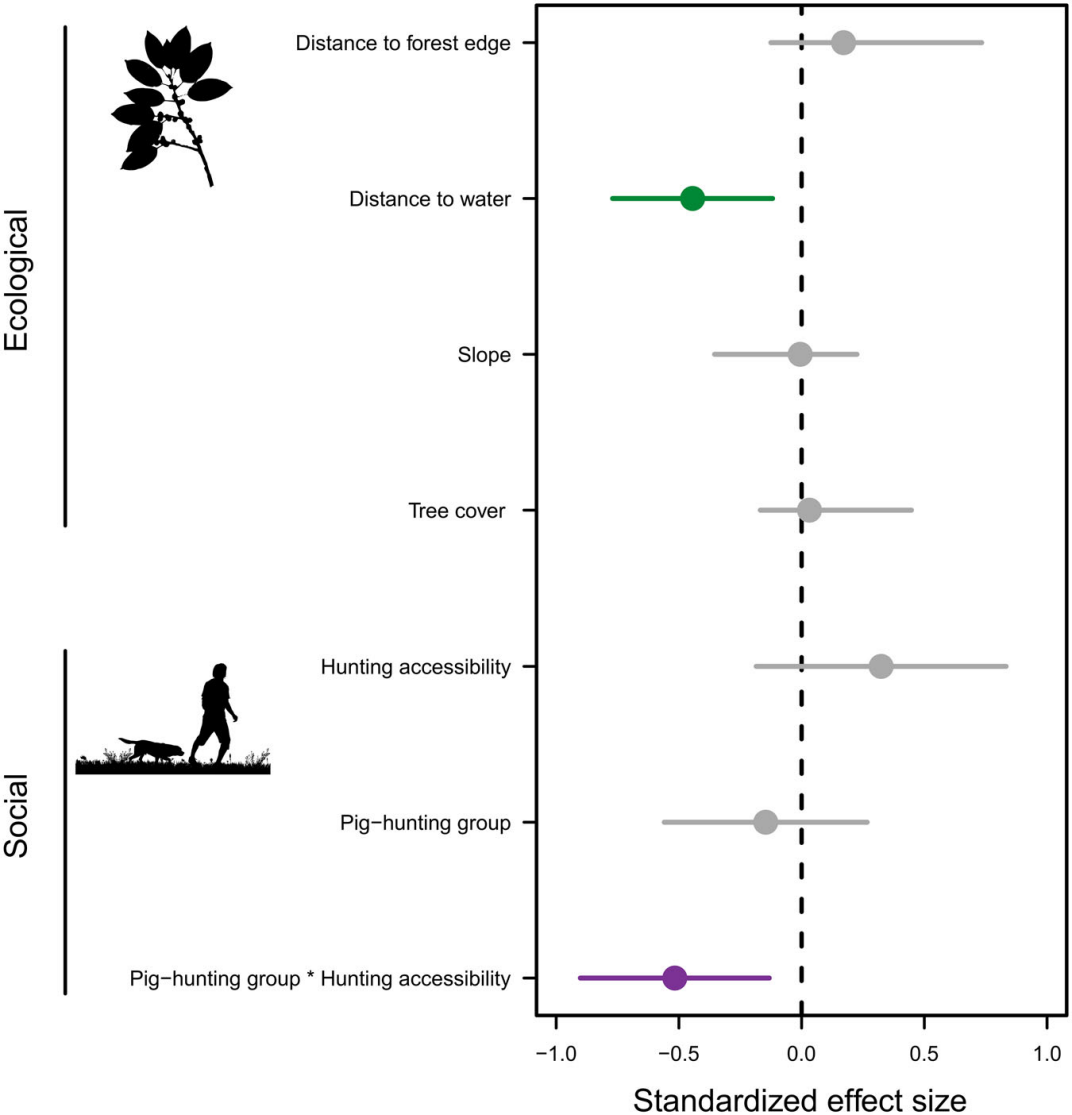
Standardized effect size median values and 95% confidence intervals (CI) for all occupancy covariates in model average of top models within <2 ΔAICc of the top model. Pig occupancy is associated with all covariates in the model-averaged model; however, the influence of covariates with 95% CI that do not overlap with zero can be considered significant. Silhouettes are made available for use through a CC0 1.0 license (top) and a standard license agreement (bottom).

All top-ranking occupancy models included both socio-cultural and ecological covariates (Table 1). Pig occupancy was associated with four ecological covariates in competitive models: distance to forest edge, distance to water, slope, and tree cover. Pig occupancy was significantly negatively associated with distance to water in the model average of top models (Fig. 1). Occupancy probability was associated with three socio-cultural covariates in competitive models: hunting accessibility, the proportion of the district population composed of the KDM or Iban group (hereafter, “pig-hunting group”), and their interaction (Table 1). Pig occupancy was significantly negatively associated with the interaction between pig-hunting group and hunting accessibility in the model average of top models (Fig. 1). Elevation and protected area status were not present in the top models.

**Table 1:** Top-ranked occupancy models.

Model	W	AICc	ΔAICc
p(eff) + ψ(dWat) + ψ(dFE) + ψ(PHgrp) + ψ(Access) + ψ(PHgrp*Access)	0.066	4226.8	0.00
p(eff) + ψ(dWat) + ψ(PHgrp) + ψ(Access) + ψ(PHgrp*Access)	0.059	4227.0	0.23
p(eff) + p(NT) + ψ(dWat) + ψ(dFE) + ψ(PHgrp) + ψ(Access) + ψ(PHgrp*Access)	0.035	4228.1	1.30
p(eff) + ψ(dWat) + ψ(PHgrp) + ψ(Access) + ψ(TC) + ψ(PHgrp*Access)	0.033	4228.2	1.40
p(eff) + ψ(dWat) + ψ(dFE) + ψ(PHgrp) + ψ(Access) + ψ(TC) + ψ(PHgrp*Access)	0.031	4228.3	1.50
p(eff) + p(NT) + ψ(dWat) + ψ(PHgrp) + ψ(Access) + ψ(PHgrp*Access)	0.031	4228.3	1.52
p(eff) + ψ(dWat) + ψ(dFE) + ψ(PHgrp) + ψ(Access) + ψ(slp) + ψ(PHgrp*Access)	0.025	4228.7	1.94

Occupancy models within <2 ΔAICc of the top ranked model; “p” indicates detection probability and “ψ” indicates occupancy probability. “Model” shows the variables present in the model, “W” indicates model weight, “AICc” indicates corrected Akaike Information Criterion, and ΔAICc indicates the difference in the AICc between the model and the top model. [Covariate abbreviations are: Access = Hunting accessibility; dFE = distance to forest edge; dWat = distance to water; eff = sampling effort; NT = non-tree vegetation cover; PHgrp = Pig-hunting group; TC = Tree cover; slp = Slope; PHgrp*Access = Interaction of pig-hunting group and hunting accessibility].

### Interaction between Indigenous pig-hunting group and hunting accessibility

**Fig. 2:**
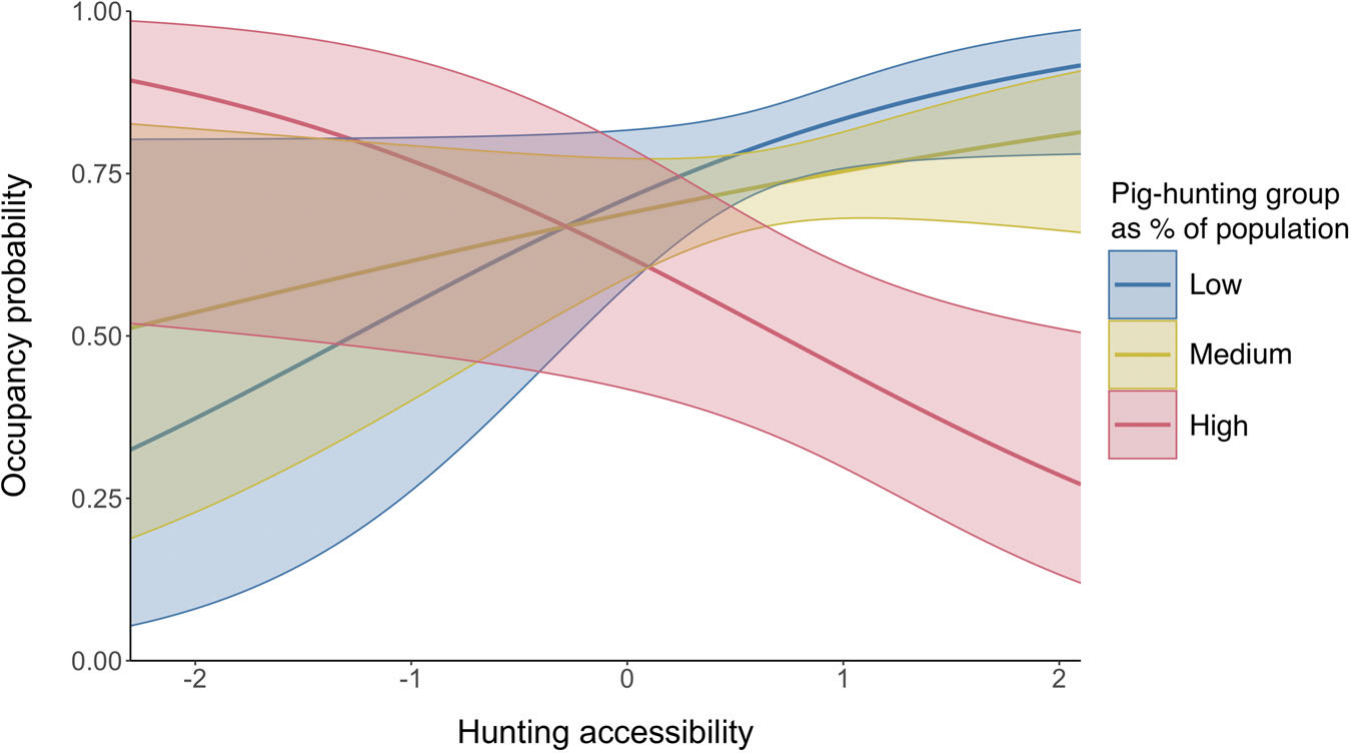
Plot showing a prediction, based on the model-averaged results, of the interaction between pig-hunting group and hunting accessibility, while holding other covariates constant. Bands show 95% confidence intervals. Percent is standardized.

The interaction between pig-hunting group and hunting accessibility significantly negatively influenced pig occupancy in the model-averaged model (Fig. 1). At low levels of hunting accessibility, a prediction based on model-averaged results showed that pig occupancy was positively associated with a high level of pig-hunting group (Fig. 2). At high levels of hunting accessibility, the prediction based on model-averaged results showed that pig occupancy was positively associated with medium and low levels of pig-hunting group (Fig. 2).

### Estimated occupancy values and detection associations

Our results show relatively high occupancy estimates of bearded pigs across most sites within our study area in Sabah and Sarawak (Table 2). Predicted average bearded pig occupancy across all study sites from our model-averaged occupancy models was 0.70 (95% CI [0.57, 0.81]). Predicted average bearded pig occupancy per site ranged from 0.92 (95% CI [0.74, 0.97]) at Danum Valley Conservation Area to 0.35 (95% CI [0.21, 0.52]) at Crocker Range National Park (Table 2). Notably, relatively high and low estimated pig occupancies were present in Sabah and Sarawak, in protected and unprotected areas, in primary and secondary forests, and in hill and montane forests.

**Table 2:** Estimated occupancy probabilities for bearded pigs across study sites in Malaysian Borneo.

Site	Pred. occupancy	95% CI
Crocker Range National Park	0.35	[0.21, 0.52]
Danum Valley Conservation Area	0.92	[0.74, 0.97]
Gunung Mulu National Park	0.51	[0.32, 0.71]
Hose Mountains	0.66	[0.37, 0.86]
Lower Kinabatangan Wildlife Sanctuary	0.85	[0.76, 0.92]
Madai Baturong	0.90	[0.78, 0.96]
Maliau Basin Conservation Area	0.76	[0.64, 0.84]
Malua Forest Reserve	0.77	[0.61, 0.87]
Pulong Tau National Park	0.74	[0.61, 0.83]
SAFE Project	0.73	[0.57, 0.85]
Sapulut	0.37	[0.23, 0.54]
Silabukan	0.80	[0.69, 0.88]
Sipitang	0.77	[0.66, 0.86]
Tabin Wildlife Reserve	0.78	[0.64, 0.87]
Tawau Hills Park	0.79	[0.64, 0.88]
Ulu Baram	0.61	[0.46, 0.74]
Ulu Padas	0.70	[0.60, 0.79]
Ulu Trusan	0.78	[0.68, 0.85]

Occupancy probability estimates were based on data from sampling in 2010, 2012, and 2014, prior to the outbreak of African Swine Fever in 2021. Occupancy estimates for most sites drew from data from a single year. Occupancy probability predictions are based on model-averaged results.

Bearded pig detection was associated with two covariates in the model average of top models: sampling effort and non-tree vegetation cover. Detection was significantly positively associated with sampling effort in the model average of competitive models (standardized effect size = 0.296, 95% CI [0.202, 0.389]). Non-tree vegetation cover was present in only two top models (Table 1), and pig detection was not significantly associated with non-tree vegetation cover in the model average of top models (standardized effect size = −0.009, 95% CI [−0.122, 0.045]).

## Discussion

Our results provide robust, quantitative evidence that socio-cultural and ecological factors underpin the spatial distribution of a large-bodied game species. By demonstrating the importance of socio-ecological drivers in wildlife distribution modeling for this species, our approach goes beyond many conventional modeling frameworks that consider ecological factors in isolation or that use broad indices of human footprint. Furthermore, we quantitatively show that Indigenous pig hunting is potentially compatible with high bearded pig occupancy. Our data point to the importance of context-specific Indigenous and local management; sustainable, locally-led Indigenous pig hunting may be well-suited to low accessibility areas and may be feasible in some high accessibility areas with thresholds collaboratively agreed upon by Indigenous and state decision makers. Additionally, our occupancy predictions show relatively high pig occupancies across many sites, from camera surveys prior to the 2020–2021 ASF outbreak, suggesting that a full bearded pig recovery from ASF could help sustain Indigenous pig hunting and robust bearded pig populations.

Socio-cultural covariates were predictors of bearded pig occupancy, highlighting the relevance of cultural practices for wildlife distributions. While conceptual models of socio-ecological systems are continuing to develop^11^, and urban ecology has embraced human demographics and cultural practices in wildlife distribution assessments^32,33^, we assert that it is critical to normalize more fully the integration of socio-cultural practices into wildlife ecology and conservation. Published accessibility maps, census data, and government-collected social data provide opportunities for such quantitative integration with wildlife data from camera traps, surveys, acoustic data recorders, citizen science datasets, and integrated databases^34,35^. In our study context, social landscapes and ecological landscapes share important intersections. For example, hunter movements are strongly associated with landscape characteristics across Malaysian Borneo^10^. Our results build on this link by showing that the interaction between hunting accessibility and pig-hunting cultural groups is associated with a game species response.

Pig-hunting group and hunting accessibility showed a strong interaction, with important lessons for socio-ecological theory and wildlife management. At low levels of hunting accessibility, predicted pig occupancy was positively associated with a predicted high level of pig-hunting group. At high levels of the hunting accessibility metric (which includes population density), predicted pig occupancy was negatively associated with a predicted high level of pig-hunting group. However, in high accessibility areas, predicted pig occupancy was positively associated with predicted medium and low levels of pig-hunting group. These findings highlight the importance of population density, landscape topography, and human culture in jointly shaping sustainable Indigenous hunting in context-specific ways. The role and relevance of ethnicity and religion, including cultural practices, in theoretical and empirical studies of wildlife distribution have been too often overlooked. Robust cultural traditions have important implications for conservation value formation^36^, and social practices shape wildlife utilization patterns across the globe^6,37,38^. Our findings validate this theoretical consideration by empirically demonstrating the connections between pig-hunting cultural group, hunting accessibility, and bearded pig occupancies in Malaysian Borneo.

In our study region, ethnic identification is linked to religion, leading to additional cultural implications for bearded pig hunting. The majority of KDM and Iban communities identify as Christian (although a minority are Muslim), and the vast majority of Malays identify as Muslim^18^. These ethno-religious identifications add an additional layer of group identity to pig hunting practices and dietary choices. In fact, food practices rooted in religion and ethnicity are so strong in our study area that a “pig line” has been recorded in Sarawak between Muslim fishing communities along the coast and Christian pig-hunting communities in the interior^39^. Our results show that two Indigenous, Christian hunting traditions—by Iban and KDM groups—shape bearded pig distributions, and may continue to be compatible with bearded pig conservation. While ethno-religious traditions have been linked to harvest of wild animals and plants^40–42^, relatively few studies have quantitatively explored these connections. Therefore, by showing ethno-religious influences on bearded pig occupancy, our findings make a compelling case for the wider relevance of cultural factors on wildlife utilization patterns generally.

Our study focused on the influence of socio-ecological factors on occupancy, and did not include density estimates within the scope of our analysis. Bearded pigs have a number of population states of varying densities under different environmental conditions^43^, presumably resulting in varying levels of resilience to hunting pressure. In areas with plentiful food resources and high pig densities, bearded pig resilience to hunting could be high; indeed, during resource-rich mast fruiting periods, female bearded pigs can give birth to 10–30 piglets in a single year^25^. In Sabah, older pig hunters recall pig mass movements more than younger hunters^6^; this pattern may point to fewer large-scale Dipterocarp mast fruiting events accompanied by bearded pig herding behavior^43,44^. Additionally, in contemporary Malaysian Borneo, oil palm plantations provide year-round food subsidies to bearded pigs in many areas^6,31^; for example, in one mixed landscape, bearded pig tracks were found in 80% of oil palm transects adjacent to forest^23^. Therefore, the relationship between high bearded pig densities and hunting deserves further study, and could hold important implications for context-specific hunting management.

The outbreak of African Swine Fever (ASF) has led to the collapse of bearded pig populations across Sabah, and is a threat to populations in Sarawak^27^. ASF case fatality rates of 47.7 to 100% have been recorded in wild and domestic pigs^45,46^. Our site-specific findings—from data collected prior to the ASF outbreak—therefore provide a critical baseline of bearded pig occupancy in a number of areas within Malaysian Borneo. At the time of publication, bearded pig occupancies in a number of our sites are almost certainly much lower than the estimates reflected here, as a result of widespread local population collapses due to ASF^27^. As populations recover, further research can compare future bearded pig occupancy relative to these baselines for specific areas as part of ongoing monitoring efforts for this IUCN-listed Vulnerable species^21^. For example, ongoing camera trap surveys across protected areas could help managers and decision makers assess the utility of hunting policies, movement control orders, law enforcement, and other regulatory mechanisms that could assist in bearded pig recovery. Protecting and expanding existing parks, wildlife reserves, and wildlife corridors may also provide connectivity and habitat for numerous bearded pig sub-populations, providing contexts for pig populations to safely recover.

Our results support context-specific management of bearded pigs in Sabah and Sarawak, Malaysian Borneo. Average estimated occupancy probability for bearded pigs across all sites was relatively high, at 0.70 (95% CI [0.57, 0.81]). However, spatial variation in predicted occupancy, which varied widely across field sites (0.35–0.92), suggests a range of management needs for bearded pig populations, which are known to have widely varying movement patterns and population growth rates that correspond to different population states^43^. Bearded pig natural history fits many characteristics of a generalist species—e.g., dietary flexibility, broad habitat use, and high dispersal ability^25,31^, and indeed our results showed high estimated occupancy probabilities in primary, secondary, protected, unprotected, hill, montane, and lowland forests in our study. However, the species still seems to require forest cover for safety, thermoregulation, and nesting^23,27,47^. Widespread habitat destruction in recent decades has led to losses of large portions of its range and habitat in Borneo as well as in Peninsular Malaysia and Sumatra^28^. As such, management strategies that preserve and protect contiguous forest areas are of critical long-term importance.

Our results show a significant, negative relationship between bearded pig occupancy and distance to water. This finding suggests the importance of proximity to water for high bearded pig occupancies. Our data do not allow us to conclude which aspect of proximity to water is particularly important for bearded pig populations, but there are several potential hypotheses. One possibility is that nearby water availability provides ready access to thermoregulation, as bearded pigs need to wallow regularly to cool themselves in warm tropical conditions^27^. Another hypothesis is that *Ficus* sp. (fig) trees are more abundant or of greater diversity in riparian areas in our study area, providing a steady food source to sustain pig populations. Figs are thought to be a key food resource for bearded pigs during inter-mast intervals between Dipterocarp fruit pulses^48^. Additionally, some *Ficus* species are particularly prominent in riparian areas in Southeast Asia^49,50^, suggesting that this may hold true for our study areas. Management policies could consider protecting areas with Dipterocarps, *Ficus* sp. trees, *Fagaceae* sp. (oak) trees, and other food sources for bearded pigs, thereby providing variation in fruiting cycles that can sustain bearded pigs through lean periods^43,48^. Large protected areas with abundant Dipterocarps may also provide one of the last contexts for conserving the unique migratory ecology of bearded pigs, which are thought, historically, to have traveled in very large herds for hundreds of kilometers tracking supraannual mast fruiting bounties^44^. Large tracts of protected forest in Borneo and Sumatra are likely the only places remaining with sufficiently high masting activity, intact migration corridors, low hunting risk, large bearded pig populations, and reduced influence of oil palm fruit subsidies – together potentially establishing conditions for long-range bearded pig nomadic movements. In Borneo, continued tri-lateral cooperation between the governments of Brunei, Malaysia, and Indonesia through the Heart of Borneo initiative will be essential to protect large intact forest amidst infrastructure development, logging, mining, and oil palm expansion^51,52^.

Finally, our results provide quantitative support for the potential sustainability of Indigenous pig hunting practices in Malaysian Borneo. Our results indicate that in both high and low hunting accessibility areas, there may be pathways for sustainable bearded pig hunting. The predicted positive relationship, based on our model results, between pig occupancy and Indigenous pig-hunting group in low accessibility areas suggests that there likely continue to be pathways for sustainable Indigenous bearded pig hunting in rural and remote areas. In low accessibility areas, Indigenous bearded pig hunting may be compatible with goals of biocultural conservation, such as provision of dietary and cultural benefits to KDM and Iban communities and conservation of bearded pig populations^6,16^. As such, our study lends support to customary Indigenous pig hunting rights, which have been compatible with sustainable bearded pig populations for thousands of years^19^. In high accessibility areas, the predicted positive association between pig occupancy and medium and low levels of pig-hunting group may suggest the need for adaptive, context-specific management that incorporates a certain threshold of hunting as a valid land use^53^. In particular, the population density of a specific area and the level of hunting pressure in that area may be important considerations for management^10^. Critically, hunting thresholds and other management techniques should be determined collaboratively by local Indigenous and state leaders in ways that are sensitive to long-standing Indigenous hunting practices, local cultural and dietary needs^6^, area-specific ecological and management considerations, and bearded pig population trends. Our finding tracks with other studies highlighting opportunities for integration of Indigenous harvesting practices and sustainable management of game species^54–56^.

After bearded pig populations recover to pre-ASF levels, we recommend preserving Indigenous pig hunting rights in specific areas while also ensuring long-term bearded pig population stability in protected areas. Prior to the ASF outbreak, bearded pig hunting was legal and common in both Sabah and Sarawak outside of protected areas and, in the case of Sabah, with a hunting license^16,57–59^. Our results suggest that in low accessibility areas, high pig occupancies may be compatible with greater local participation in Indigenous hunting. In high accessibility areas, high pig occupancies may be compatible with moderate local participation in Indigenous hunting. In particular, forest-oil palm mixed landscapes, which are generally in high accessibility areas^10,29^, are known to support high levels of bearded pig utilization^23,31^. Regulated hunting in and around industrial and small-scale oil palm plantations could provide pest control benefits in plantations while also offering meat provision and cultural benefits for KDM and Iban hunters^6,16^. Additionally, limiting or eliminating the sale of wild meat could help preserve Indigenous hunting for subsistence and cultural purposes while also reducing unsustainable hunting pressure on bearded pig populations. Balancing pig populations and hunting is likely possible, given relatively high pig occupancy estimates across most of our study sites and high bearded pig reproductive capacity^25^. However, further research on cultural practices and values, Indigenous hunting practices (in collaboration with Indigenous groups), pig densities, up-to-date records of hunting rates, and ongoing monitoring of pig populations are needed before specific regulations could be most accurately developed by Indigenous and state leaders.

Nuanced solutions will be critical so that Indigenous communities in Sabah and Sarawak can continue to sustainably hunt bearded pig populations, as they have for millennia^19,22^. Critically, policies should allow for the flourishing of Indigenous stewardship practices, which have been connected to local conservation ethics^6^. For integrated biocultural conservation goals, it is critical that KDM and Iban groups are free to preserve the cultural and dietary benefits of bearded pig hunting, while also limiting hunting to sustainable levels for bearded pig population recovery and stability^6^. Our results highlight the tensions and opportunities of these twin goals.

## Methods

### Study region and data collection

**Fig. 3:**
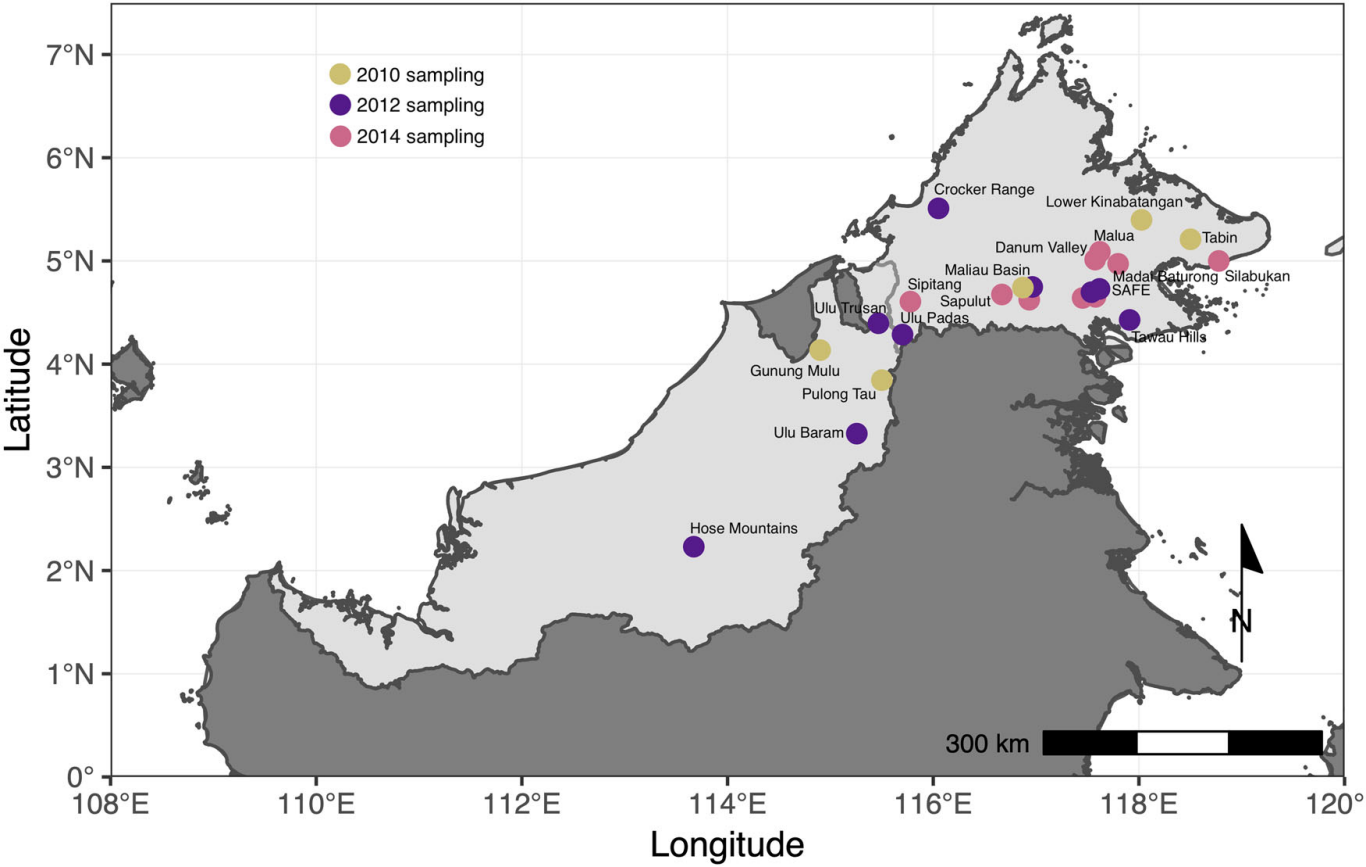
Map of study area. Location of wildlife camera surveys conducted in 2010, 2012, and 2014 across Sabah and Sarawak, Malaysian Borneo. Labels show the names of the 18 sites, which are marked with dots, color-coded by year of the survey. For two sites, multiple locations within the site and multiple years of surveying are shown by clusters of dots.

We collated bearded pig detections and non-detections from 18 camera trap surveys conducted in 2010, 2012, or 2014 across the Malaysian Bornean states of Sabah and Sarawak (Fig. 3). We followed institutional guidelines for research and obtained local research permissions from the Economic Planning Unit - Malaysia, Forest Department Sarawak, Sabah Biodiversity Centre, Sabah Forestry Department, Sarawak Biodiversity Centre, and Sarawak Forestry Corporation (permit numbers listed in Acknowledgements).

From these surveys, we integrated data from a total of 295 camera trap locations across 18 sites, comprising 10,462 bearded pig detections across 25,755 trap days. Camera surveys were designed to target multiple mammal species and were therefore suitable for detecting bearded pigs, one of the most common mammals in many camera surveys in our study area^60,61^. We spatially filtered camera locations to ensure at least 1 km between locations. For studies using paired cameras at each sampling location, we applied a random number generator to randomly select one camera from the pair. Camera trap surveys were carried out within lowland, hill, and montane primary forests (within protected areas) and previously logged lowland, hill, and montane secondary forests (including both protected and non-protected areas)^62^. Landscape accessibility across the study area varied considerably; our survey data included low and high accessibility areas^10^. Within arrays, cameras were active for varying lengths of time (Supplementary Table 1).

### Ecological covariates

We chose covariates that we hypothesized to be strong predictors of bearded pig occupancy (Table 3). Ecological covariates (extracted from Earth Engine Data Catalog) included in analyses were distance to water^63^, distance to forest edge^64^, protected area status^65^, elevation^66^, slope^66^, and tree cover^67^ (Table 3, Supplementary Table 2). To match ecological variables with the camera trap data, we either used the closest year of ecological data or an average of multiple years. We removed highly correlated continuous covariates until Pearson’s correlation coefficients among all pairs were less than |0.6|^68,69^. To allow for ready comparison among covariate coefficients, we standardized all ecological covariates, except for protected area status (which is a factor).

**Table 3:** Social and ecological covariates included in occupancy models.

Model covariate	Hypothesized relationship with occupancy	Covariate type
Hunting accessibility	—	social
KDM or Iban Indigenous group	—	social
Hunting accessibility^a^Indigenous grp.^1^	—	social
Distance to water (m)	—	ecological
Distance to forest edge (m)	+	ecological
Protected area status	+	ecological
Elevation (m)	+	ecological
Slope (deg)	—	ecological
Tree cover (%)	+	ecological

KDM is a locally-used abbreviation for the Kadazandusun-Murut Indigenous group.

1 The ^a^represents an interaction effect. An interaction effect occurs when the level of one covariate influences the effect of another covariate on the dependent variable, which in this case is pig occupancy.

### Social covariates

We chose social variables that we hypothesized to influence bearded pig distributions (Table 3). The social covariates included in the models were: pig-hunting group (proportion of the district population composed of the predominant Indigenous pig-hunting group), hunting accessibility, and their interacting effects (Table 3, Supplementary Table 2). For the pig-hunting group covariate, we used published census data to calculate the proportion of the district population composed of the KDM peoples in Sabah and the Iban peoples in Sarawak^6,14,18^.

Our study was not set up to explore hunting pressure directly, e.g. through separate covariates for raw numbers of total hunters per district and physical accessibility of the landscape. Instead, we used a published metric—which we refer to as “hunting accessibility”—that shows landscape accessibility adjusted for coarse estimates of plausible hunter density^10^. A higher metric score indicates higher hunting accessibility, and vice versa^10^. Despite measures of physical accessibility incorporated into the hunting accessibility metric, the metric was not highly correlated with any of our ecological covariates at a level above |0.4|^68^. While this metric also accounts for the relative human population in a given area^10^, it is unable to capture cultural influences within the local populations engaging in hunting in that area, which is influenced by ethnicity and religion^6^. Alongside hunting accessibility, we included the covariate “pig-hunting group” (see above), thereby incorporating Indigenous, Christian pig-hunting practices into our models. We also included an interaction term for pig-hunting group and hunting accessibility, which tests whether the level of one of these covariates influences the impact of the other on pig occupancy. As with ecological covariates, we standardized social covariates to allow for ready comparison of coefficients.

### Detection covariates

We used percent of non-tree vegetation cover^67^ and effort as our predictors for detection.

Non-tree vegetation cover can block a pig from view and thereby inhibit the ability of a given camera to detect a passing pig. Including effort, using the package ‘unmarked’^70^, as a detection covariate in occupancy models allowed us to correct for variable sampling effort, per trap and occasion window. As with ecological and social covariates, we standardized detection covariates to allow for ready comparison of coefficients.

### Occupancy modeling approach

We fitted one single-season occupancy model for all the data from 2010, 2012, and 2014. However, given the long-distance movements for which the bearded pig is known^24,25,44^, detections of bearded pigs cannot be considered independent and their populations cannot be considered closed^71^. Therefore, model-estimated occupancy results for species in this situation should be interpreted as probability of site use rather than true occupancy^72,73^.

We ran models using the packages ‘unmarked’^70^ and ‘camtrapR’^74^ in R version 3.6.0^69^. We used a seven-day sampling occasion window, corrected for sampling effort per trap and per occasion within the ‘camtrapR’ package^74^. We then used the ‘dredge’ function^75^ in R version 3.6.0^69^ to identify top models according to Akaike’s Information Criterion values, adjusted for small sample sizes (AICc). Models within <2 ΔAICc of the top model were considered to be competing models^76^. We model-averaged competing models. We used the function ‘confint()’ to calculate 95% confidence intervals for coefficients^69^. To visualize the interaction between pig-hunting group and hunting accessibility, we created an interaction plot prediction (repeated 267 times) using packages ‘ggplot2’ and ‘AICcmodavg’^77,78^.

### Supplementary information

Supplementary Information

## Data Availability

The census data and environmental variable data used in this study are publicly available (for links, see Supplementary Table 2). Data sharing for the camera trap data is not applicable to this study, as no new camera data were collected for this study. Requests for camera data may be made to the authors of the original camera studies for which those data were collected.
