# Supplementary material for: Socio-ecological factors shape the distribution of a cultural keystone species in Malaysian Borneo
Source: NPJ Biodivers. 2023 Feb 17;2:4. doi: 10.1038/s44185-022-00008-w (PMC11332217; doi:10.1038/s44185-022-00008-w)
Supplement: Supplementary file 1 — Supplementary Information [file 44185_2022_8_MOESM1_ESM.pdf]

Kurz et al. 2023, Supplementary Information

Supplemental Abstract. Abstract translation in Malay (Bahasa Malaysia).

Gabungan bersama antara faktor biofizikal dan sosio-budaya telah membentuk taburan biodiversiti global, namun masih hanya sedikit kajian secara kuantitif telah dilaksanakan bagi menilai pengaruh landskap sosial dan ekologi terhadap taburan hidupan liar. Kami berusaha untuk menentukan sama ada kovariat sosial dan kovariat ekologi dapat mempengaruhi taburan bagi spesies kunci, iaitu babi hutan (Sus barbatus). Dengan menggunakan set data sebanyak 295 lokasi perangkap kamera dan 25,755 jumlah perangkap hari daripada 18 tapak lapangan selama tiga tahun di Sabah dan Sarawak, Borneo Malaysia, kami menyatukan model-model penghunian yang menggabungkan kovariat sosio-budaya dan kovariat ekologi, yang dihipotesiskan boleh mempengaruhi penghunian babi hutan. Kami dapati bahawa kovariat sosio-budaya dan kovariat ekologi juga termasuk dalam semua model penghunian yang kompetitif. Selain itu, kami menemui bukti kuantitatif yang menyokong hak pemburuan-babi orang asal: Jangkaan penghunian babi adalah berkait secara positif terhadap jangkaan kumpulan tahap tinggi pemburuan-babi orang asal di dalam kawasan kebolehcapaian rendah, dan jangkaan penghunian babi juga berkait secara positif terhadap jangkaan kumpulan tahap sederhana dan rendah pemburuan-babi orang asal di kawasan kebolehcapaian tinggi. Keputusan ini mencadangkan agar populasi babi hutan di Borneo Malaysia harus diselaraskan dengan strategi khusus-konteks, yang dapat mempromosikan hak pemburuan-babi orang asal. Kami juga menyediakan maklumat asas yang penting tentang tahap-tahap penghunian babi hutan sebelum wabak demam babi Afrika atau dikenali sebagai African Swine Fever (ASF) pada tahun 2020-2021, yang menyebabkan kebimbangan sosial dan ekologi selepas berlaku kematian babi hutan dengan jumlah yang sangat tinggi di Borneo.

Supplementary Table 1. Number of cameras and trapping period length for all study sites. Note that the total number of cameras listed here is from the original study data. Thus, the total number of cameras listed here is larger than in the main text, as we dropped cameras during the analysis (e.g. for geographic filtering).

Study site	Number of cameras	Trapping period (days)
Crocker Range National Park	35	144
Danum Valley Conservation Area	42	60
Gunung Mulu National Park	17	113
Hose Mountains	19	91
Lower Kinabatangan Wildlife Sanctuary	66	146
Madai Baturong	11	58
Maliau Basin Conservation Area	27	125
Malua Conservation Area	42	60
Pulong Tau National Park	16	154
SAFE Project 2012	194	104
SAFE Project 2014	79	32
Sapulut	30	121
Silabukan	20	151
Sipitang	25	127
Tabin Wildlife Reserve	133	91
Tawau Hills Park	77	225
Ulu Baram	17	58
Ulu Padas	19	104
Ulu Trusan	20	39

Supplementary Table 2. List of variables included in study, along with metadata. All variables listed here were occupancy covariates, except non-tree vegetation, which was a detection covariate. Sampling effort was also a detection covariate and is not listed here; sampling effort was calculated within the package 'unmarked'.

Variable	Units	Spatial Res.	Source	Citation	Dataset or Dataset Description URL
Tree cover	Percent	250m	MOD44B: MODIS/Terra Vegetation Continuous Fields Yearly L3 Global 250 m SIN Grid V006	Dimiceli, C. et al. MOD44B MODIS/Terra Vegetation Continuous Fields Yearly L3 Global 250m SIN Grid V006. NASA EOSDIS Land Processes DAAC. https://doi.org/10.5067/MODIS/MOD44B.006 (2015).	https://doi.org/10.5067/MODIS/MOD44B.006
Non-tree vegetation	Percent	250m	MOD44B: MODIS/Terra Vegetation Continuous Fields Yearly L3 Global 250 m SIN Grid V006	Dimiceli, C. et al. MOD44B MODIS/Terra Vegetation Continuous Fields Yearly L3 Global 250m SIN Grid V006. NASA EOSDIS Land Processes DAAC. https://doi.org/10.5067/MODIS/MOD44B.006 (2015).	https://doi.org/10.5067/MODIS/MOD44B.006
Elevation	Meters	30m	SRTM Digital Elevation Data 30m (NASA JPL Version 3.0)	Farr, T. G. et al. The shuttle radar topography mission. *Rev. Geophys.* https://doi.org/10.1029/2005RG000183 (2007).	https://doi.org/10.1029/2005RG000183
Slope	Degrees	30m	Derived from SRTM Digital Elevation Data 30m (NASA JPL Version 3.0)	Farr, T. G. et al. The shuttle radar topography mission. *Rev. Geophys.* https://doi.org/10.1029/2005RG000183 (2007).	https://doi.org/10.1029/2005RG000183
Distance to any water	Meters	30m	Derived from JRC Yearly Water Classification	Pekel, J.-F., Cottam, A., Gorelick, N. & Belward, A. S. High-resolution mapping of global surface water and its long-term changes. *Nature* **540**, 418-422 (2016).	https://doi.org/10.1038/nature20584
Protected area status	Category (Polygons)	NA	World Database of Protected Areas	UNEP-WCMC & IUCN. Protected planet: the world database on protected areas (WDPA). https://www.protectedplanet.net (2019).	https://developers.google.com/earth-engine/datasets/catalog/WCMC_WDPA_current_polygons
Distance to nearest forest edge	Meters	30m	Derived from CIFOR	Gaveau, D. L. A., Salim, M. & Arjasakusuma, S. Deforestation and industrial plantations development in Borneo. Center for International Forestry Research (CIFOR). https://doi.org/10.17528/CIFOR/DATA.00049 (2016).	https://doi.org/10.17528/CIFOR/DATA.00049
Hunting accessibility	Meters	90m	See citation	Deith, M.C.M & Brodie, J.F. Predicting defaunation: accurately mapping bushmeat hunting pressure over large areas. *Proc. Royal Soc. B.* https://doi.org/10.1098/rspb.2019.2677 (2020).	https://doi.org/10.1098/rspb.2019.2677
Pig-hunting group	Percent	District	Malaysian Department of Statistics	Malaysia Department of Statistics. Taburan penduduk dan ciri-ciri asas demografi. https://www.mycensus.gov.my/index.php/ms/produk-banci/penerbitan/banci-2010/664-taburan-penduduk-dan-ciri-ciri-asas-demografi-2010 (2011).	https://www.mycensus.gov.my/index.php/ms/produk-banci/penerbitan/banci-2010/664-taburan-penduduk-dan-ciri-ciri-asas-demografi-2010
